# 

**Published:** 1890-10

**Authors:** 


### CONTENTS.

*EDITORIALS.* PAGE

Heredity of Tuberculosis, . . . . . . . . . . 433

Surgery the Opprobrium of Medicine, . . . . . . . 434

The Opprobrium of Old School Medicine, . . . . . . 437

Dr. Frank Kraft, . . . . . . . . . . . . . 437

*MISCELLANEOUS.*

Mono-pharmacy. Translated by E. A. P., . . . . . . . . 438

Duration of Action of Antidotes of the Principal Remedies. F. H. Lutze, M. D., . . . . . . . . . . . . . 439

The Dynamic Forces of the Homœopathic Remedies, . . . 457

Eczema. Wm. Steinrauf, M. D., . . . . . . . . 462

Puerperal Convulsions. W. A. Yingling, M. D., . . . . . 464

Puerperal Convulsions. Chas. P. Beaman, M. D., . . . . . 467

Novel Cases of Poisoning. S. L., . . . . . . . . . 469

Ulcers. A. McNeil, M. D., . . . . . . . . . . 470

Ulcers, . . . . . . . . . . . . . . . . . 472

Some Provings of Merc-Iod. CM, and Kali-Iod. 2d. Ella M. Tuttle, M. D., . . . . . . . . . . . . . 473

Brief Contribution to the Elucidation of Syphilis. John Hall, M. D., 473

Verifications. Wm. Jefferson Guernsey, M. D., . . . . . 475

Errors in Dr. T. F. Allen's *Encyclopedia.* T. F. Allen, M. D., . . 477

Notes from Past Meetings of the Lippe Society, . . . . . 478

